# Buffering of Segmental and Chromosomal Aneuploidies in *Drosophila melanogaster*

**DOI:** 10.1371/journal.pgen.1000465

**Published:** 2009-05-01

**Authors:** Per Stenberg, Lina E. Lundberg, Anna-Mia Johansson, Patrik Rydén, Malin J. Svensson, Jan Larsson

**Affiliations:** 1 Department of Molecular Biology, Umeå University, Umeå, Sweden; 2 Computational Life Science Cluster (CLiC), Umeå University, Umeå, Sweden; 3 Department of Mathematics and Mathematical Statistics, Umeå University, Umeå, Sweden; 4 Department of Statistics, Umeå University, Umeå, Sweden; European Molecular Biology Laboratory, Germany

## Abstract

Chromosomal instability, which involves the deletion and duplication of chromosomes or chromosome parts, is a common feature of cancers, and deficiency screens are commonly used to detect genes involved in various biological pathways. However, despite their importance, the effects of deficiencies, duplications, and chromosome losses on the regulation of whole chromosomes and large chromosome domains are largely unknown. Therefore, to explore these effects, we examined expression patterns of genes in several *Drosophila* deficiency hemizygotes and a duplication hemizygote using microarrays. The results indicate that genes expressed in deficiency hemizygotes are significantly buffered, and that the buffering effect is general rather than being mainly mediated by feedback regulation of individual genes. In addition, differentially expressed genes in haploid condition appear to be generally more strongly buffered than ubiquitously expressed genes in haploid condition, but, among genes present in triploid condition, ubiquitously expressed genes are generally more strongly buffered than differentially expressed genes. Furthermore, we show that the 4^th^ chromosome is compensated in response to dose differences. Our results suggest general mechanisms have evolved that stimulate or repress gene expression of aneuploid regions as appropriate, and on the 4^th^ chromosome of *Drosophila* this compensation is mediated by Painting of Fourth (POF).

## Author Summary

Although deletion heterozygotes and chromosomal aneuploidies have been used in genetic studies for decades, the relationships between chromosome doses and transcript outputs have been difficult to unravel. In other words, the effects of copy changes on the regulation of entire chromosomes or large chromosomal domains are largely unknown. Hence, we studied these relationships in *Drosophila* using microarrays prepared from flies with a dosage series of chromosomal domains and a dosage series of the 4^th^ chromosome. We observed significant buffering of expressed genes, i.e., on average they were expressed at >50% of wild-type levels when present in single copies instead of two copies (the normal complement of diploids). This buffering was also seen to be much stronger for differentially expressed genes than ubiquitously expressed genes. Our findings therefore support the presence of chromosome-wide buffering mechanisms. In addition, we found evidence of a chromosome-specific protein POF-mediated mechanism in the buffering of the 4^th^ chromosome. Overall, our results suggest that a general buffering system acts on most genes present as single copies due to deletions or chromosome losses. Further work on gene buffering effects should make substantial contributions to our understanding of genome-wide gene regulation.

## Introduction

The effects of deficiencies, duplications or chromosome losses (e.g. somatic elimination) on the regulation of whole chromosomes and large chromosome domains are largely unknown, although the gene dose at most specific loci generally has little effect on the development of *Drosophila*. A useful scale for assessing the magnitude of aneuploidies that can be accommodated in the *D. melanogaster* genome without loss of viability was provided by Bridges, who divided the genome into 102 numbered divisions, based on cytological analysis of polytene chromosomes [1]. Deletions extending over more than one of these 102 divisions (which have estimated sizes of 800–1500 kb [2], with a median length of 1114 kb according to Flybase annotation) are generally lethal [3]. However, there are a few known exceptions of longer, non-lethal deletions, such as *Df(2L)H* and *Df(3L)Vn*, which span <2.8 Mb and <1.7 Mb, respectively [4]. A general rule in *Drosophila* is that viability and fertility are reduced when having a single copy of ∼1% of the genome, but raising this proportion to ∼3% is lethal [3]. Hence, segmental aneuploidy-induced mortality could be explained by altered levels of gene expression within the aneuploid region, leading to an overall disturbance of gene networks [5]. However, it has been suggested that a reduced dose of any region will cause a general effect on expression of the genome and since most effects are negative in correlation to dose this is sometimes referred to as the “inverse dosage effect” [6].

Intuitively, we may expect transcript levels of genes within an aneuploid region to correlate directly with the gene dosage. However, some reports have suggested that functional autosomal dosage compensation, also known as the “buffering” effect, may occur, e.g. activities of proteins expressed from genes present in three copies, due to segmental trisomy, were found to be very similar to wild type levels in several early dosage studies [7]–[9]. Since these early studies of correlations between expression levels and gene doses relied mainly on enzyme assays (although transcript levels of single genes were sometimes measured), dose responses at the transcription level were unclear, due to the potential effects of post-transcriptional processes. However, indications of buffering effects have also been obtained in recent dose response studies using genome-wide approaches [10]–[12]. For example, ∼1.4 fold differences in mRNA levels associated with three-fold differences in gene doses in a *Drosophila* autosomal region have been found in microarray analyses [10],[13], substantially lower than the expected 3-fold differences in the absence of compensation. It should be noted that genome-wide studies inevitably include analyses of non-expressed genes and genes expressed at sub-detectable levels; two groups of genes that will inevitably be scored as fully compensated (i.e. as being expressed at apparently wild type levels) and thus influence the mean calculated buffering effect.

Convincing reports of chromosomal dosage compensation have hitherto only been observed in the sex chromosomes, leading to the general conclusion that this mechanism exclusively equalizes transcription between the two sexes, and compensates for the difference in the expression of sex chromosomes in relation to autosomes [13]–[17]. However, we have previously demonstrated another chromosome-wide regulatory system in *Drosophila* [18],[19], in which the Painting of fourth (POF) protein binds specifically to the 4^th^ chromosome and together with heterochromatin protein 1 fine-tune the expression of genes in this chromosome [18],[20]. Further, flies with a single 4^th^ chromosome are viable and fertile, like flies that have a single X-chromosome, but in marked contrast to flies that have lost any other autosome. These and other observations have prompted suggestions that a dosage compensation mechanism may act upon the 4^th^ chromosome [21].

To gain insight into the expression consequences upon chromosome 4 aneuploidies and also segmental aneuploidies in general, we have made a detailed genome-wide analysis of gene expression in aneuploidy regions in *Drosophila*. Using expression microarrays of haplo-4, diplo-4 and triplo-4 flies, we show that expressed genes are significantly compensated, and that the compensation in haplo-4 flies is dependent on POF. Furthermore, we show that segmental aneuploidy regions are slightly buffered and this buffering is suggested to be at a general level and not mainly caused by a single gene feed-back regulation. Overall, the presented results suggest that general mechanisms exist to stimulate and repress gene expression.

## Results

### Expressed Genes in Segmental Aneuploidies Are Buffered

To study the effect of gene dose on gene expression total RNA was prepared from flies with the following genotypes: heterozygous for *Df(2L)J-H*, *Df(2L)ED4470* and *Df(2L)ED4651* deletions; heterozygous for the *Dp(2;2)Cam3* duplication; monosomic for chromosome 4 (*4/0*); trisomic for chromosome 4 (*4/4/4*); and wild type controls (where *Df* and *Dp* indicate deficiency and duplication, respectively). Each of these genotypes, the lengths of the affected sequences, and the respective numbers of affected genes are listed in Table S1. Three biological replicates representing each genotype were hybridized to Affymetrix *Drosophila* v2 arrays, and the resultant raw data were normalized and summarized using RMA [22]. Global effects in the genome outside of our used aneuploidies can potentially influence data analysis and normalisation. We therefore analysed the raw data prior to any normalisation and could not detect any major global effects. Global effects are further discussed in Text S1 and Figure S1. Non-expressed genes and genes with expression levels that are sub-detectable in the micro-array analysis will be scored as fully compensated when the aneuploids are compared to the wild type. Including these genes inevitably shifts the average closer to wild type expression levels, potentially leading to over-estimates of any buffering effect. Therefore, cut-offs for genes with correctly measured expression levels were determined by plotting transcription levels in mutants against wild type expression levels (Figure S2). The resulting plots showed that aneuploidy effects were only detected for genes with wild type expression levels >6 (log_2_-scale). In all arrays we then removed the genes with wild type expression values below 6 and renormalized the expression values. In this normalisation, a constant was added to all the mutant array expression values to ensure that the total genomic expression matched that of the wild type. The average expression relative to wild type was then measured for all of the expressed genes within each aneuploid region.

**Figure 1:**
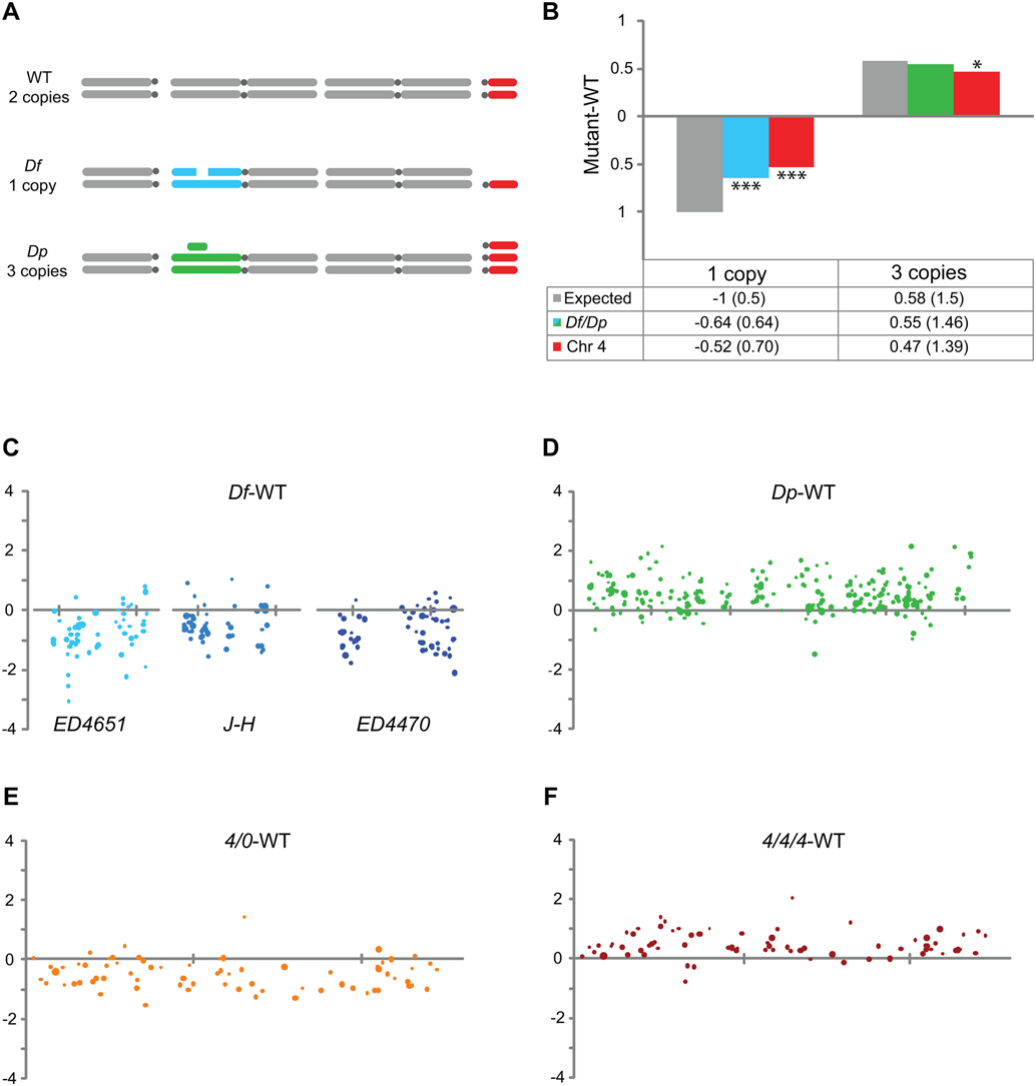
Genes in segmental and chromosomal aneuploidies are buffered. (A) Schematic illustration of the genotypes used in our study. (B) The y-axis and the table below the diagram shows log_2_ values (with non-logarithmic values given in parentheses) of expression differences between deficiency (indicated in blue), duplication (green), haplo-4 and triplo-4 (both red) mutants compared to wild type. The deficiencies and haplo-4 conditions are significantly buffered compared to the expected expression output (grey). The asterisks indicate significantly different values from expected values. (C–F) Expression levels of individual genes in relation to wild type levels, plotted against chromosomal position for the three deficiencies (C), the duplication (D), haplo-4 (E) and triplo-4 (F). The tick marks correspond to 500 kb and the individual gene expression levels in wild type are indicated by the size of the dots (the higher the gene expression level the larger the dot). Only genes with expression levels >6 are shown.

Genes within the *Df* regions were significantly buffered (one sample Wilcoxon test, p<<0.001), since they were expressed at 64% of wild type levels, compared to the 50% expression level expected under the naïve assumption of regulatory independence (Figure 1B). This buffering effect was weaker than those observed in previous studies [10]–[12], and we hypothesized that this difference was mainly due to our exclusion of non- and weakly-expressed genes. This speculation was confirmed, since the buffering levels in our pre-cut-off data were similar to previously reported values (data not shown). However, it is important to note that it is still not known whether weakly-expressed genes are actually buffered, and if so to what degree.

The effects of the aneuploid regions are shown in plots of moving median expression ratios along the chromosome arms in Figure S3. A significant buffering effect was detected in the *4/0* flies (one sample Wilcoxon test, p<<0.001), of similar strength to that observed in the *Df* flies (Mann-Whitney U test, p = 0.21). A triploid region (*Dp*) in the *4/4/4* background also showed a buffering effect, with a slight decrease in expression (146% compared to the expected 150%), although this was not significant (one sample Wilcoxon test, p = 0.079). *Df(2L)J-H*/+ flies are viable also in *4/0* background and there was no significant difference in the effects of the *Df(2L)J-H* deficiency in wild type compared to *4/0* backgrounds (Mann-Whitney U test, p = 0.28). However, the entire 4^th^ chromosome was significantly compensated in *4/4/4* flies (139% compared to the expected 150%, one sample Wilcoxon test, p = 0.015). Chromosome 4 will be discussed in more detail below.

### The Buffering Effect Is Approximately Normally Distributed

**Figure 2:**
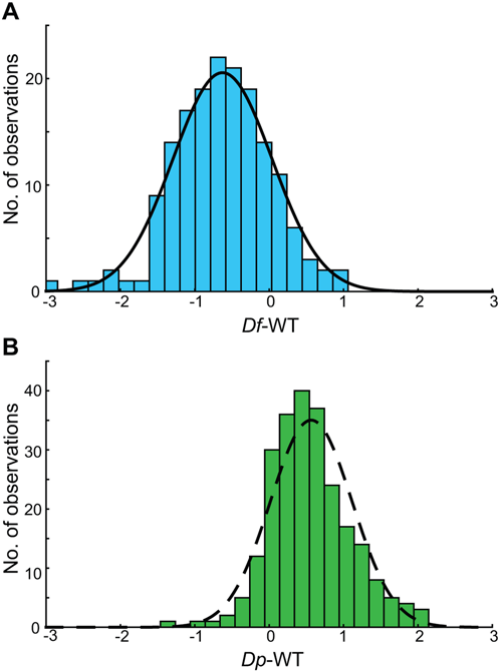
Expression differences between deficiencies and wild type are approximately normally distributed. (A) The distribution of expression differences between all three deficiencies and wild type, with a normal distribution curve superimposed. (B) The distribution of expression differences between the duplication and wild type is not normally distributed.

The observed buffering effect could have been caused by either the feed-back regulation of individual genes or a more general buffering mechanism. However, if it was mainly caused by the former, the distribution of differences in expression levels between the *Df* and wild type genotypes would probably be highly skewed, since most genes would be expected to be expressed at close to 50% of wild-type levels, while the expression of a few genes would be buffered to varying degrees. Instead, the expression differences were approximately normally distributed (Shapiro-Wilk's W test, p = 0.20) around a mean of 64% wild-type expression (Figure 2A). In contrast, the *Dp* genotypes showed no significant buffering effects, and the differences between their expression levels and wild-type levels were not normally distributed (Shapiro-Wilk's W test, p = 0.0030, Figure 2B). This could mean that any potential buffering system for genes when they are present in three copies is less evolved than when they are present in one copy.

### Ubiquitously Expressed Genes in *Df* Regions Are Not Buffered

**Figure 3:**
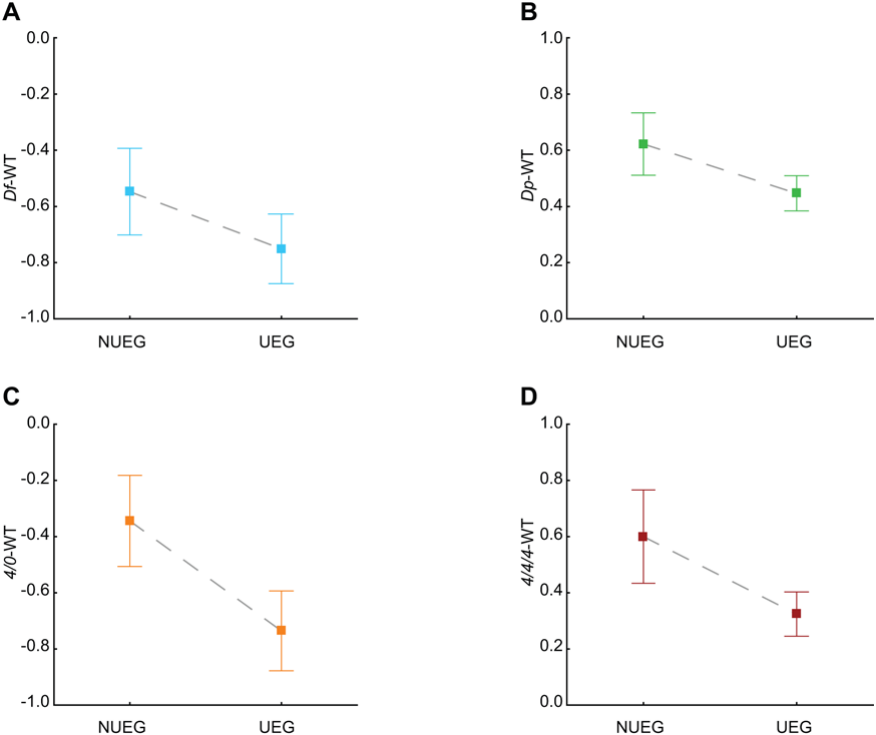
The buffering of segmental and chromosomal haploids mainly acts on non-ubiquitously expressed genes. Mean differences in NUEG and UEG gene expression, relative to wild type, observed in (A) deficiencies (n_NUEG_ = 91, n_UEG_ = 75), (B) the duplication (n_NUEG_ = 138, n_UEG_ = 102), (C) haplo-4 (n_NUEG_ = 39, n_UEG_ = 33), and (D) triplo-4 (n_NUEG_ = 39, n_UEG_ = 33). Squares indicate mean values and whiskers indicate 95% confidence intervals.

We then asked whether the observed buffering effect correlated with any particular class of genes. No correlations were found between the buffering effect and expression levels, except for a weak relationship in *4/4/4* flies (Spearman correlation, p = 0.032, Figure S4). Neither were there any correlation between the buffering effect and gene length (data not shown). However, a clear correlation was found between buffering and ubiquitously expressed genes (UEGs) (Figure 3), here defined as genes expressed at levels >6 in all 12 tissues present in the FlyAtlas database [23]. The UEGs were significantly less buffered than non-ubiquitously expressed genes (NUEG) in the *Df* and *4/0* flies (Mann-Whitney U test, p = 0.021 and p = 0.00045, respectively, Figure 3A and 3C). Conversely, the NUEGs were significantly less buffered in the *Dp* and *4/4/4* flies (Mann-Whitney U test, p = 0.038 and p = 0.0017, respectively, Figure 3B and 3D). Thus, UEGs appear to be only buffered when present in three copies.

### Chromosome 4 Is Compensated in Response to Dose Reductions

**Figure 4:**
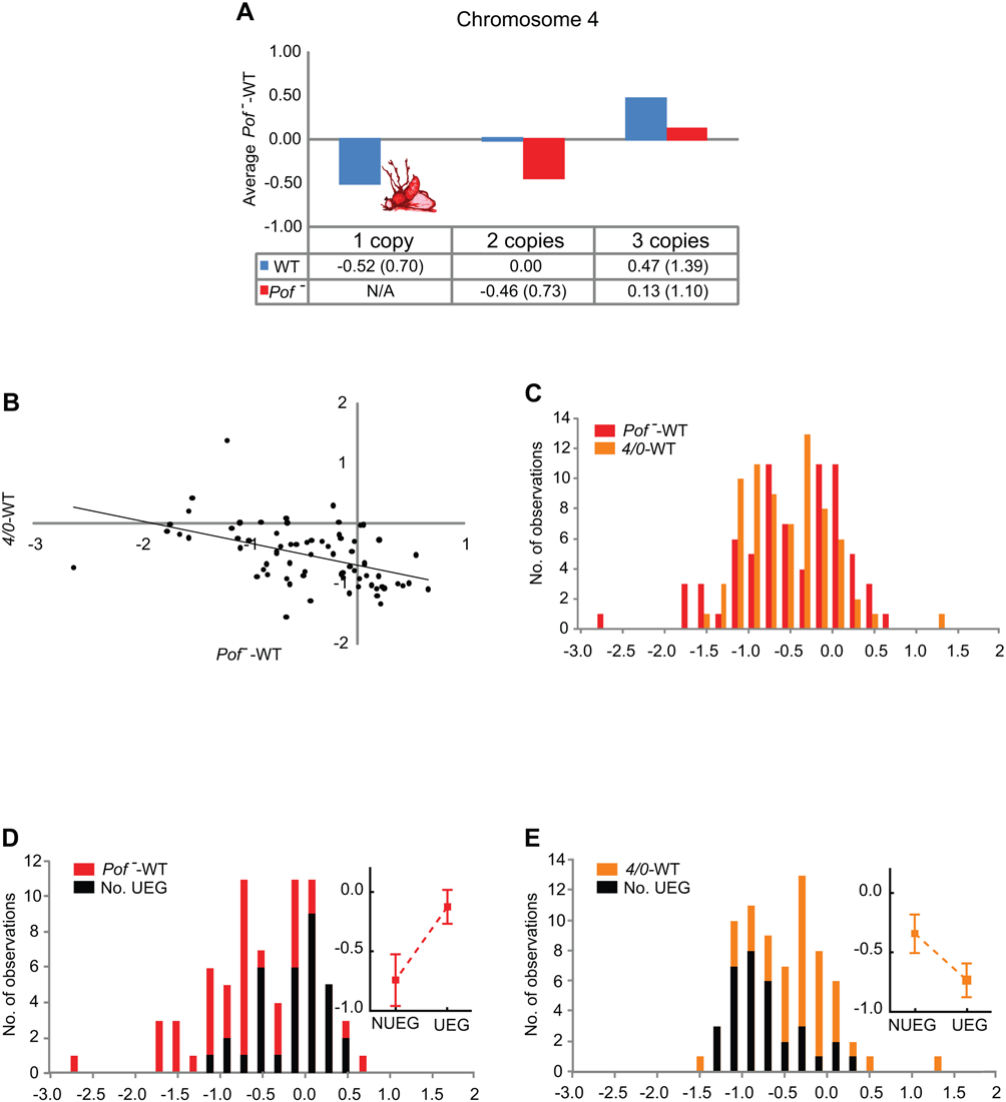
The 4^th^ chromosome is compensated by POF when present in single dose. (A) Average differences in expression in genes of the 4^th^ chromosome in wild type and *Pof^-^* mutants as affected by chromosome 4 dose. The y-axis and the table below the diagram show log_2_ values for the expression differences, and non-logarithmic values are shown within parentheses. The haplo-4 condition is lethal in the *Pof^-^* mutant. (B) Correlation plot of haplo-4 – WT and *Pof^-^* – WT differences in expression values (log_2_ scale) demonstrating POF-mediated compensation of the 4^th^ chromosome. The regression line is indicated. (C) Distribution of haplo-4 – WT and *Pof^-^* – WT differences in expression values. Note the twin-peak distribution. (D) Distribution of *Pof^-^* – WT differences in expression levels, with the total numbers of genes and UEGs in red and black, respectively. The mean difference, as plotted in Figure 3, is superimposed. (E) Distribution of haplo-4 – WT differences in expression levels, with total numbers of genes and UEGs in orange and black, respectively. Note that the distribution of UEGs differs between (D) and (E).

As shown in Figure 1, chromosome 4 is compensated in response to altered dose. Compensation of the 4^th^ is slightly higher but not significantly different from compensation in segmental aneuploidies (deficiencies). We have previously shown that the protein POF specifically stimulates gene expression on the 4^th^ chromosome, and that *Pof* is essential for the survival of *4/0* flies [18]. Hence, we constructed expression arrays from *Pof* mutants with two or three copies of the 4^th^ chromosome (no arrays of mutants with a single copy could be made, since haplo-4 flies do not survive without POF). As seen in Figure 4A, POF always stimulated expression, regardless of the 4^th^ chromosome copy number. Strikingly, there was also a clear negative linear correlation between the differences in expression, relative to the wild type, between the *4/0* and *Pof* mutants (Figure 4B, Pearson correlation, r = −0.48, p<<0.001). This implies that the level of compensation in *4/0* flies is inversely proportional to the level of expression change in *Pof* mutants. Thus, we conclude that the compensation observed in *4/0* is directly mediated by POF. Moreover, the distributions of the buffering effects in *4/0* and *Pof* mutants were not normal (Shapiro-Wilk's W test, p = 0.014 and p = 0.014 respectively), but rather displayed two clear peaks (Figure 4C). Both of these data sets therefore appear to contain data on one group of strongly affected genes and one that is almost unaffected. The unaffected groups consisted mainly of NUEGs in *4/0* and UEGs in *Pof* mutants, whereas the strongly affected groups were mainly composed of UEGs in *4/0* and NUEGs in *Pof* mutants (Figure 4D and 4E).

### Loss of *Pof* Causes Reductions in Levels of Chromosome 4 Gene Expression in Testes

The high expression of POF in the testes and the strong relationship between POF and dosage compensation prompted us to examine the role of POF in the testes. In order to understand the role of POF we performed immunostainings for POF and immunofluorescens localisation of a *P[Pof.EYFP]* transgenic constructs in male testes. The results are presented and discussed in Figure S5 and Text S1.

**Figure 5:**
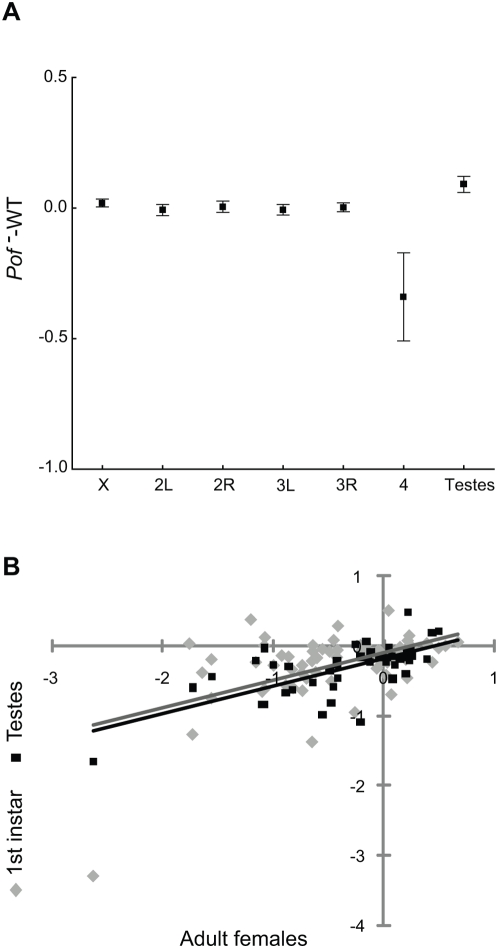
POF stimulates the same set of genes in three different stages. (A) Mean change of gene expression for each chromosome arm and for a defined group of testes expressed genes (N_testes_ = 865). Squares indicate the mean values and whiskers indicate 95% confidence intervals. (B) Correlation plots of *Pof^-^* – WT differences in expression levels (log_2_ scale) obtained for testes tissue (black) and 1^st^ instar larvae (grey) plotted against corresponding differences for adult females. Regression lines are indicated.

Expression arrays were then used to assess the influence of POF on transcription in the dissected testes (*Pof* mutants and wild type control), and the results clearly showed that POF mainly altered the expression of genes in the 4^th^ chromosome (Figure 5A). We then tested whether the expression levels of testes-specific genes were altered in *Pof* mutants. We did observe a weak effect on these genes, although unexpectedly the expression was higher in *Pof* mutants (104%, one sample Wilcoxon test, p<<0.001), which we hypothesise could be caused by delayed spermatogenesis.

Average reductions in expression levels were found to be similar in *Pof* mutant adult female, testes and first instar larvae tissues (first instar data from [18], Kruskal-Wallis ANOVA by Ranks, p = 0.18). The effect on individual genes was also seen to be linearly correlated (Figure 5B, three pair-wise Pearson correlations, r = 0.51–0.68, p<<0.001), and thus we conclude that the effect of POF on chromosome 4 genes is the same in all three of these tissues.

## Discussion

Segmental and chromosomal aneuploids have been used in genetic studies for several decades. However, the transcriptional effects of aneuploidies have been far from fully elucidated, partly because the exploration of genome-wide responses requires genome-wide analysis, which has only been possible since the advent of reliable microarray techniques, such as those used in the presented study. The results obtained show there was significant buffering of genes in a haploid region, although the degree of buffering was much lower than previously reported. In addition, the buffering appeared to be far more efficient for differentially expressed genes than for ubiquitously expressed genes. The mechanism responsible for buffering on the major autosome arms is still unknown, but our results show that compensation for haploidy of the 4^th^ chromosome is mediated by the protein POF. We have used the term *buffering* for the autosomes (for which the mechanism is unknown) and *compensation* for the fourth chromosome since in the latter case we can show that this depends on POF.

### Expressed Genes in a Haploid Region Are Buffered

Previous studies on the relation between chromosome dose and transcript level response suggest the existence of buffering effects [10]–[12]. The effect is dramatic, a three-fold difference in gene dosage, obtained using the *Df* and *Dp* genotypes examined here, were found to be associated with ∼1.4 fold differences in transcript levels, rather than the expected 3-fold differences [10],[13]. It is important to note that mRNA levels have been measured in most genome-wide expression studies, and thus it is still unclear whether the observed effects are due to transcriptional differences or post-transcriptional effects.

Using all our data we found a buffering effect of similar strength to those previously reported (a 3-fold difference in gene dosage resulted in 1.5 fold differences in transcript levels). However, we also found that expression can only be reliably measured for genes with relative expression levels >6, and when we only analyzed these genes we found a less dramatic, but still significant buffering effect of deficiencies. In contrast, when expressed genes were analyzed, no buffering effects in responses to duplication were detected. Hence, gene dosage reductions (but not apparently increases in dosage) can be compensated for by buffering, when all the expressed genes are considered.

### The Observed Buffering Suggests a General Effect

What causes the observed buffering effect? We can consider two plausible models to explain this. First, the calculated buffering effect may be a consequence of a more or less complete feed-back regulation of a subset of genes. Secondly, the observed buffering is mainly caused by a general increased expression of the genes uncovered by the *Df*. The obtained expression values for *Df*-WT were normally distributed and centred on a mean expression value of 0.64 (Figure 2). The normality of the distribution suggests that the observed buffering effect was general, and thus that individual gene feed-back regulatory mechanisms (which would probably have yielded a skewed distribution) were not primarily responsible for the calculated mean effect. Hence, the results from the *Df* indicate that the buffering system is general, and that the variation around the mean is mainly caused by array noise.

Two possible general buffering mechanisms could also be envisioned. Firstly, a monosomic region could be “sensed” and actively targeted by compensating protein complexes, similar to those described for the male X-chromosome and the 4^th^ chromosome in *Drosophila* [15],[18]. Alternatively, there could be feedback regulation of a few individual genes, and stimulated expression could result from high local concentrations of transcription-stimulating factors and/or “spread” from the nuclear environment of a single region. The mechanism for the suggested general buffering effect is likely to be a mixture of events at different levels which remains to be unravelled.

### Aneuploidies Cause Opposite Responses in Differentially and Ubiquitously Expressed Genes

We examined whether the observed buffering was correlated to expression levels. This is a reasonable assumption since in the two known chromosome-wide regulatory systems in *Drosophila*, the MSL mediated dosage compensation and POF mediated regulation of the 4^th^ chromosome, there is a relation between protein binding to genes and expression levels. In the case of dosage compensation, MSL binding is correlated to expressed genes but not to expression levels [24],[25]. However, to a large extent MSL binding reflects the expression levels in young embryos and the binding is then for most genes stable throughout development [26]. On the other hand, POF binding to the 4^th^ chromosome is linearly correlated to gene expression levels [20]. Even though POF binding to genes is directly correlated to gene expression levels we find no correlation of buffering effects to gene expression levels.

We also examined whether differences in the normal regulatory patterns of genes affect their degree of buffering, by dividing the set of studied genes into ubiquitously expressed genes (UEGs) and non-ubiquitously expressed genes (NUEGs), then comparing their buffering levels. The results indicated that UEGs can be repressed, but not stimulated (as seen in the *Dp* and *Df* genotypes, respectively). The UEG expression levels are probably primarily limited by their copy numbers, and thus it is not possible to further stimulate their expression when they are present as single copies. In contrast, UEGs in trisomic regions are generally more repressed than NUEGs. It should be stressed that while both UEGs and NUEGs are buffered in *Df* and *4/0* conditions, the UEGs are buffered to a much smaller extent. However, the NUEGs show no signs of buffering in *Dp* and *4/4/4* conditions.

The observed disparity between the UEGs and NUEGs must, presumably, be mainly due to regulatory differences, i.e. mechanisms have evolved that allow expression of the NUEGs to be responsive to various inducting and silencing signals, while the transcription of UEGs is steady, stable and more resistant to signal variations. The difference is even more pronounced on the 4^th^ chromosome, where the NUEGs are strongly compensated when present in single copies, i.e. in *4/0*. In addition, our data show that POF was responsible for the observed buffering of the 4^th^ chromosome, and the buffering of *4/0* was of similar strength to *Df* buffering on the major autosome arms.

POF shows strong similarities to the dosage-compensating MSL complex in evolutionary terms [19],[27], in binding profile [20] and in its function as a chromosome-wide regulator [18]. The mechanism responsible for MSL dosage compensation of the X-chromosomes is MOF-mediated hyperacetylation of H4K16. It should be noted that recent genome-wide studies suggest that MOF also acts as a more general regulator of gene expression in *Drosophila*. However, it is not known whether this general function is involved in the general dose response [28]. Nevertheless, it seems reasonable to hypothesize that the buffering effect seen in *Df* genotypes acts similarly to POF- and MSL-mediated stimulation, i.e. at the transcriptional level. We speculate that the more generally and stably expressed UEGs are less responsive to buffering functions than NUEGs, however the reasons why UEGs are less dose-responsive than NUEGs when present in three copies remains to be elucidated.

### What Causes Haplo-Insufficiency?

What causes the lethality in haplo-lethal deficiencies? It is obvious that genes with a strong influence on viability as exemplified by *Minute* (ribosomal protein encoding) genes will, when uncovered, increase the risk for lethality [29],[30]. Still, there seem to be a strong link between length of a deficiency and haplo-lethality [3]. Various models can be proposed to explain haplo-lethality caused by deficiencies that delete a large number of genes, one of which suggests that large deficiencies alter the doses of a number of genes involved in one or more genetic networks, thereby inducing lethality through a network collapse rather than alteration of the dose of any single gene [5]. Haplo-lethality could also be a consequence of the inverse dosage effect. In this model a haploid region will cause a general genome-wide stimulation since most effects are negative in correlation to dose [6]. It is difficult to predict the outcome of the inverse dose effect since the magnitude of this effect is not known. It is also unclear whether it will act on the whole genome or will be biased to the aneuploidy region as a consequence of gene clustering. Based on our data we suggest that general buffering mechanisms are present, and although no molecular mechanisms have been ascribed to buffering effects associated with segmental or chromosomal aneuploidies we speculate that increases in the length of deletions increase the pressure on the flies' buffering capacity. Hence, the plasticity of this system could compensate for monosomy up to a certain threshold, at which lethality may occur due to a collapse of buffering properties. Our study indicates the presence of buffering in *Df* but not as well in *Dp*, and a model suggesting haplo-lethality to be a consequence of buffering collapse would be consistent with such results. In general, flies tolerate duplications better than deficiencies, and our results are consistent with this general rule, since the pressure on buffering capacity seems to be weaker in the *Dp* than in the *Df* genotypes.

### The 4^th^ Chromosome Is Compensated by POF in Response to Dose Changes

We have previously shown that POF stimulates 4^th^ chromosome gene expression, and that the absence of *Pof* results in haplo-4^th^ lethality [18]. The results from the study presented here also show a significant negative linear correlation between the effects of *4/0* and the lack of *Pof*. This is intriguing, since it demonstrates that compensation of the 4^th^ chromosome is mediated by POF. Thus, we have identified the mechanism responsible for buffering of the 4^th^ chromosome. In addition, POF almost exclusively acts on NUEGs (Figure 4), although previous ChIP-chip analyses have shown POF targeting of genes to be proportional to their expression levels, regardless of whether they are UEGs or NUEGs [20]. Therefore, we hypothesize that POF binds to all expressed genes on the 4^th^ chromosome, but only the NUEGs respond to POF-mediated stimulation of expression, implying that buffering occurs after transcription initiation. Notably, both the 4^th^ chromosome and the major chromosome arms respond to buffering functions in haplo-conditions. This compensation is mediated by POF in the 4^th^ chromosome, but the mechanisms responsible for buffering of the major autosome arms are still unknown. In contrast to *Dp*, significant (repressive) buffering was also detected in *4/4/4*, possibly mediated by heterochromatin protein 1.

### POF and Testes-Specific Regulation

MSL-complex mediated, 2-fold up-regulation of the male X-chromosome is generally agreed to be the dosage compensation mechanism in somatic cells [14]–[16]. However, X-chromosome dosage compensation also occurs in the testes, where the MSL complex is not present, and to date no mechanism has been identified for this germline dosage compensation [31],[32]. However, POF is highly expressed in testes tissues [19], which along with the striking similarities between POF- and MSL-mediated chromosome-wide regulation prompted us to examine the importance of POF in the dose compensation of the X-chromosome in the testes.

The nuclear localisation of POF in many studied cell types indicates that it is associated with the 4^th^ chromosome, in accordance with results of previous ChIP-chip analyses [20],[27]. Drawing definitive conclusions about which genes, if any, POF associates with in spermatocytes is difficult (although our microarray analysis of testes tissue demonstrated the 4^th^ chromosome genes to be the main regulatory targets for POF in the male germline) due to the intense POF nuclear staining, which may mask more localised association in the spermatocyte nuclei (Text S1, Figure S5). However, there were no significant buffering effects of X chromosome genes in *Pof* mutants, so there was no evidence of POF-mediated dosage compensation in the mutant male germlines. The *Pof* mutants did show a slight increase in the expression of testes-specific genes, but this effect was minor and could have been a consequence of minor differences in spermatogenesis between our *Pof* mutant and wild type. We conclude that the average reduction in gene expression on the 4^th^ chromosome of *Pof* mutants is similar in the three studied tissue stages (adult females, testes and 1^st^ instar larvae), and that the effect on individual genes is linearly correlated.

The results shown here have implications. Deficiency screens are commonly used as a method to find genes involved in different biological pathways. Based on our results we anticipate that these screens will find UEGs more efficiently than NUEGs, although it should be stressed that the dose responses of genes with low expression levels are still not understood. The higher dose sensitivity of UEGs is supported by the dramatic effects of reductions in doses of ribosomal protein genes, as manifested in the associated Minute phenotypes [29],[30]. Notably, our simple categorization of UEGs and NUEGs classified all but one of the 61 annotated *Minute* ribosomal protein genes as UEGs. The difference in dose response between genes based on their expression also has consequences for our understanding on how chromosomal aberrations and chromosomal aneuploidies influence proper development.

## Materials and Methods

### Fly Strains Used

Flies were cultivated and crossed at 25°C in vials containing potato mash-yeast-agar. The *Df(2L)J-H/SM5* stock were obtained from the Kyoto *Drosophila* Stock Center, the *Dp(2;2)Cam3/CyO* from Bloomington, and the *Df(2L)ED4651/SM6a* and *Df(3L)4470/TM6C* from Szeged (*Df* and *Dp* indicate deficiency and duplication, respectively). *y^1^ w^67c23^* was used as wild type. *Df/+; 4/4* females were generated by crossing *Df/Bal* flies to wild type Oregon R. *Df(2L)J-H/+; 4/0* females were generated by crossing *Df(2L)J-H/SM5* to *C(4)RM sv^spa-pol^/0*. The *Df(2L)J-H/+; 4/0* offspring were isolated based on their Minute phenotype. *+; 4/0* females were generated similarly by crossing wild type to *C(4)RM sv^spa-pol^/0. Dp(2;2)Cam3/+; 4/4/4* females were generated by crossing *Dp(2;2)Cam3/CyO* to *C(4)RM sv^spa-pol^/0.* The *Dp(2;2)Cam3/+; 4/4/4* offspring were isolated based on non-Minute phenotype. The *Pof^119^; 4/4/4* females were generated similarly by crossing *Pof^119^/CyO; C(4)RM sv^spa-pol^/0* to *y^1^ w^67c23^; Pof^D119^/Pof^D119^* and the *Pof^D119^; 4/4* females were offspring from the *y^1^ w^67c23^; Pof^D119^/Pof^D119^* stock.

### Microarray Analysis

For microarray analysis total RNA was isolated using TRIzol reagent (Invitrogen) followed by a purification using RNeasy kit (Qiagen) according to the instruction by the suppliers. 10 adult females (0–24h) were used for each of three biological replicates of each genotype. For testes microarrays, 60 testes from 0–24 old males were used for each of three biological replicates of *y^1^ w^67c23^; Pof^D119^/Pof^D119^* and three replicates of *y^1^ w^67c23^* as controls. The 33 labelled cDNA probes were then hybridized to an Affymetrix *Drosophila* gene chip (version 2) and the intensity values were normalised and summarized using robust multi-array analysis (RMA) [22]. Other normalisation methods, such as MAS5, were also tested and they all gave similar results to RMA. All microarray data analyses were done using R (www.R-project.org) and the Bioconductor package [33]. The resulting data are available at http://www.ncbi.nlm.nih.gov/geo/ (Accession: GSE14517, GSE14516).

Based on expression array data in the FlyAtlas database [23] (Geo accession number: GSE7763), ubiquitously expressed genes (UEGs) were defined as genes showing expression levels of at least 6 in all of the 12 examined tissues after RMA normalization, while all other genes were defined as non-ubiquitously expressed genes (NUEGs). Testis-specific genes were defined, using the same dataset, as genes showing an expression level of ≥6 in testes and < 6 in all other tissues.

The first instar larvae data from [18] and the testis data were renormalized in the same way as the adult female data after removing all genes expressed below 6 (after RMA) in the respective wild type.

### Statistical Analysis

All statistical analyses were performed on log_2_-scaled data using Statsoft Statistica 8.0.

### Testis Preparations and Immunostaining

For whole mount immunostaining, wild type testes were dissected in PBS, fixed for 30 minutes in 4% *para*-formaldehyde in a solution containing 0.1 M Hepes, 2 mM EGTA and 1mM MgSO_4_ (pH 6.9), then stained essentially according to [34], using an anti-POF chicken polyclonal primary antibody (1∶100 dilution) followed by a pre-absorbed biotinylated Donkey anti-chicken IgY secondary antibody (1∶300, Jackson), which was detected by the brown HRP reaction (H_2_O_2_, DAB). For indirect immunofluorescence staining, testes squashes were fixed according to [35] (Protocol 5∶5). The slides were then washed in 1×PBT for 30 min, transferred to a blocking solution (0.1 M maleic acid, 0.15 M NaCl, 1% Boehringer blocking reagent) and incubated for 30 min at room temperature. The slides were incubated overnight at 4°C with a 1∶100 diluted anti-POF chicken polyclonal primary antibody, then washed for 2×10 minutes (in 0.1 M maleic acid, 0.15 M NaCl, 0.3% Tween 20), and then blocked for 30 minutes. A 1∶300 diluted donkey anti-chicken IgY conjugated with Cy3 (Jackson) was then applied as a secondary antibody prior to a further 2 h incubation at room temperature. The squashes were counterstained with DAPI (1 µg/ml) and washed for 2×10 minutes (in 0.1 M maleic acid, 0.15 M NaCl, 0.3% Tween 20) before mounting with Vectashield (Vector). Live testes squashes from young adults carrying the *P[w^+^ Pof.EYFP]* construct (*Pof* fused to enhanced yellow fluorescent protein-encoding sequence under the control of the endogenous *Pof* promoter [27]) were dissected in TB (183 mM KCl, 47 mM NaCl, 10 mM TRIS-HCl, 1 mM PMSF, 1 mM EDTA, pH 6.8) and prepared according to [35]. Preparations were examined by phase contrast, Nomarski and fluorescence microscopy under a Zeiss Axiophot microscope equipped with a KAPPA DX20C charge-coupled device camera. The images obtained were assembled and contrasted using Adobe Photoshop.

### Microarray Data

The microarray data reported in this paper have been deposited at http://www.ncbi.nlm.nih.gov/geo/ (Accession: GSE14517, GSE14516).

## Supporting Information

Figure S1 Global effects result in skewed distribution. (A) Illustration of normal (grey), shifted normal (red) and skewed (green) distributions. (B) Plotted median *Df(2L)JH/+; 4/0* minus median wild type raw individual probe level intensities (black bars). The same data with *Df(2L)JH* probes and chromosome 4 probes excluded (grey bars). Note that the slight skew in the left tail is only seen when all probes are included.
(0.17 MB PDF)

Figure S2 Measured differences in expression levels between mutants and wild type are affected by the gene expression level. In the graphs, the expression levels of genes within the affected regions are sorted according to wild type expression levels (plotted in grey), and their expression levels in the mutants are plotted as moving averages of 11 genes in blue, green, orange and red for genes within: the three deficiencies (A), the duplication (B), haplo-4 (C) and triplo-4 (D), respectively.
(0.20 MB PDF)

Figure S3 The deficiencies and the duplication mainly affect gene expression within the dose-affected regions. (A) Expression ratios of genes on chromosome 2L in *Dp(2;2)Cam3* (green), *Df(2L)ED4651* (light blue) and *Df(2L)J-H* (blue), plotted as moving medians of 41 genes against their positions on the chromosome. (B) Moving medians of gene expression rations in *Df(3L)ED4470* against gene position on chromosome 3L. The extents of the aberrations are indicated below each plot.
(0.28 MB PDF)

Figure S4 The buffering effect in segmental aneuploidies is not correlated to gene expression levels. Differences in expression levels, plotted as a function of wild type expression levels for all deficiencies - wild type (A), duplication - wild type (B), haplo-4-wild type (C) and triplo-4-wild type (D).
(0.12 MB PDF)

Figure S5 Localisation of POF in testes. (A–C) Whole mount immunostaining of testes preparations. (A) The POF antibody did not detect any signal in the *Pof* mutant males. (B) POF was detected in young primary spermatocytes, and more strongly in mature primary spermatocytes. (C) POF strongly associates with the nuclear region of the spermatid bundle, and the bundle itself. (D–F) POF strongly associates with 2–4 foci in each nucleus of the 16 young primary spermatocytes. In a later stage of spermatocyte development POF is more evenly distributed. Images taken using phase contrast (D), DAPI (E) and anti-POF antibodies (F). (G–I) Expression of POF.EYFP in unfixed primary spermatocytes, with the young and mature spermatocytes visible in the lower and upper parts of (I), respectively. Note the foci in young spermatocytes and the more dense fluorescence in mature spermatocytes, in accordance with the immunostaining results. Images presented were acquired by phase contrast (G), EYFP fluorescence (H) and merge and zoom (I). The young primary spermatocytes are shown in the lower part of (I) and the more mature spermatocytes in the upper part.
(3.64 MB PDF)

Table S1 Genotypes of the flies used in this study and the number of genes before and after the expression cut-off 6.
(0.12 MB PDF)

Text S1 Description and discussion of global effects in expression data and results on POF localisation in testes tissue.
(0.02 MB PDF)
