# Supplementary figures and images for: Buffering of Segmental and Chromosomal Aneuploidies in Drosophila melanogaster
Source: PLoS Genet. 2009 May 1;5(5):e1000465. doi: 10.1371/journal.pgen.1000465 (PMC2668767; doi:10.1371/journal.pgen.1000465)

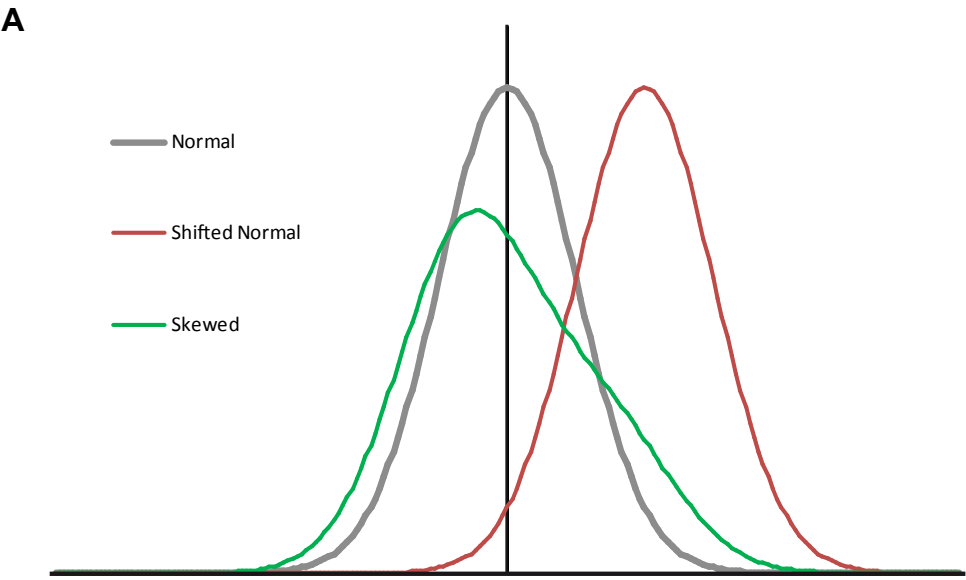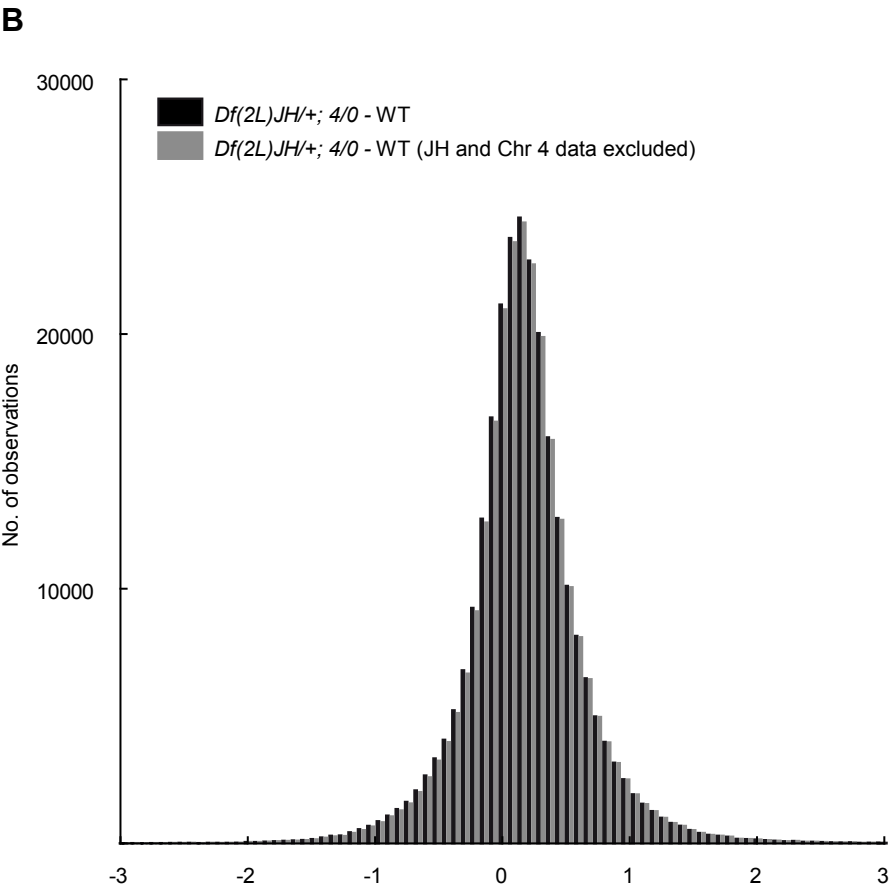

Supplement: Figure S1 — Global effects result in skewed distribution. (A) Illustration of normal (grey), shifted normal (red) and skewed (green) distributions. (B) Plotted median Df(2L)JH/+; 4/0 minus median wild type raw individual probe level intensities (black bars). The same data with Df(2L)JH probes and chromosome 4 probes excluded (grey bars). Note that the slight skew in the left tail is only seen when all probes are included. (0.17 MB PDF) [file pgen.1000465.s001.pdf]

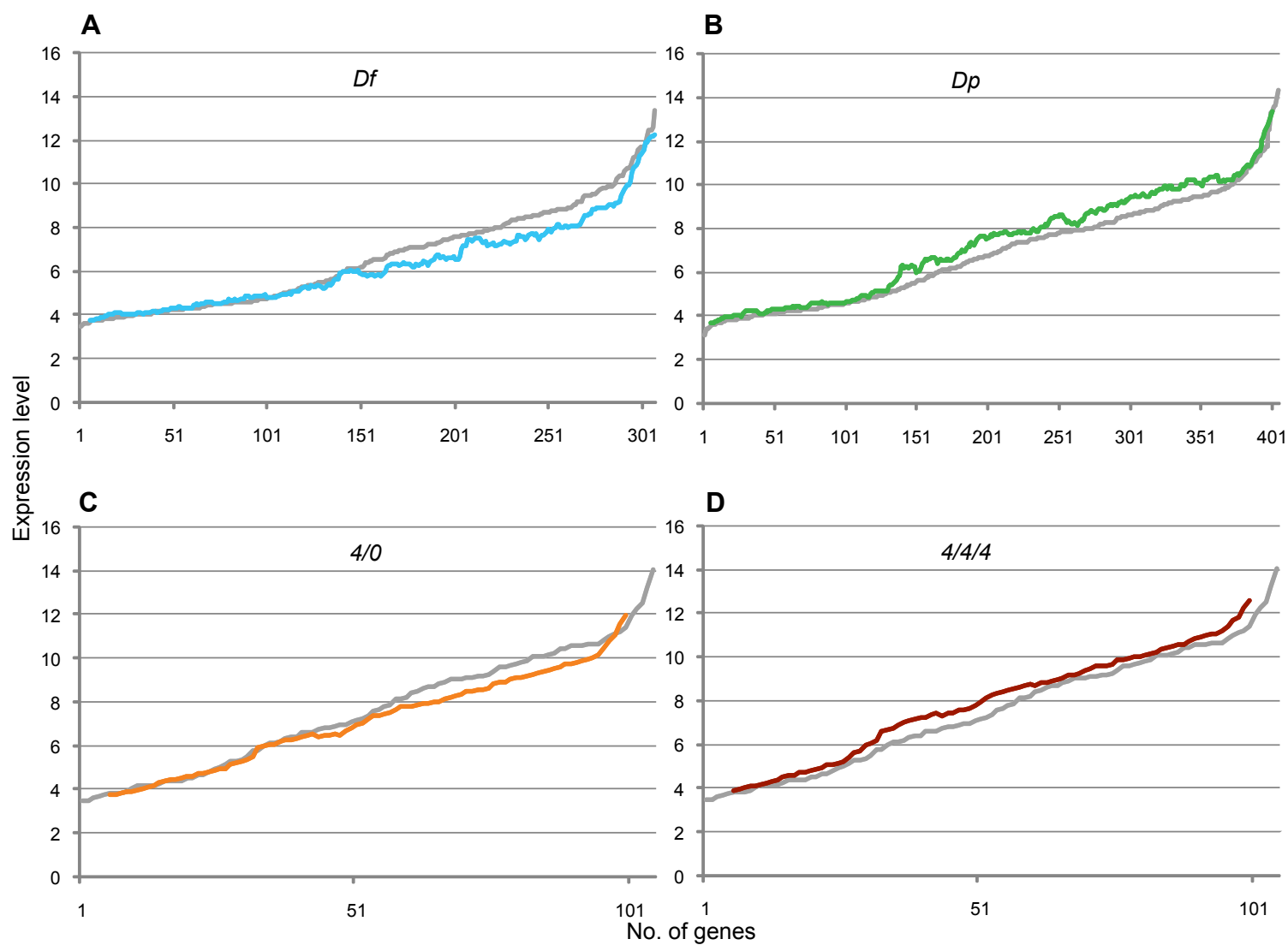

Supplement: Figure S2 — Measured differences in expression levels between mutants and wild type are affected by the gene expression level. In the graphs, the expression levels of genes within the affected regions are sorted according to wild type expression levels (plotted in grey), and their expression levels in the mutants are plotted as moving averages of 11 genes in blue, green, orange and red for genes within: the three deficiencies (A), the duplication (B), haplo-4 (C) and triplo-4 (D), respectively. (0.20 MB PDF) [file pgen.1000465.s002.pdf]

2L

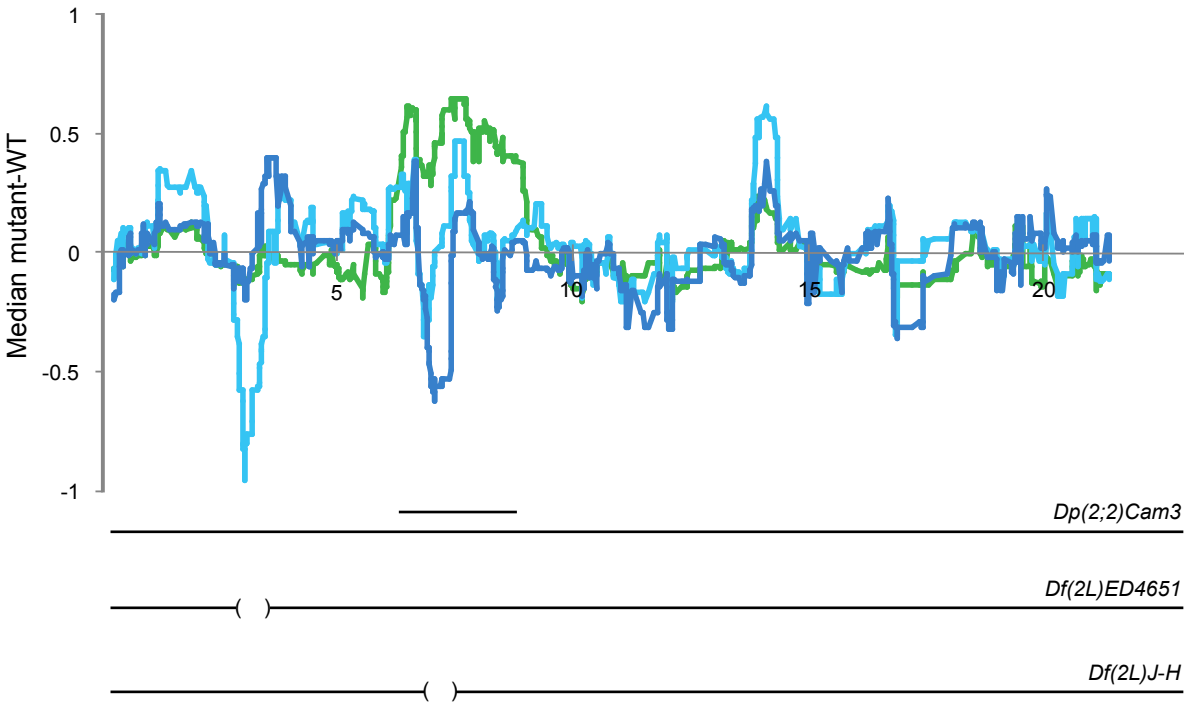

3L

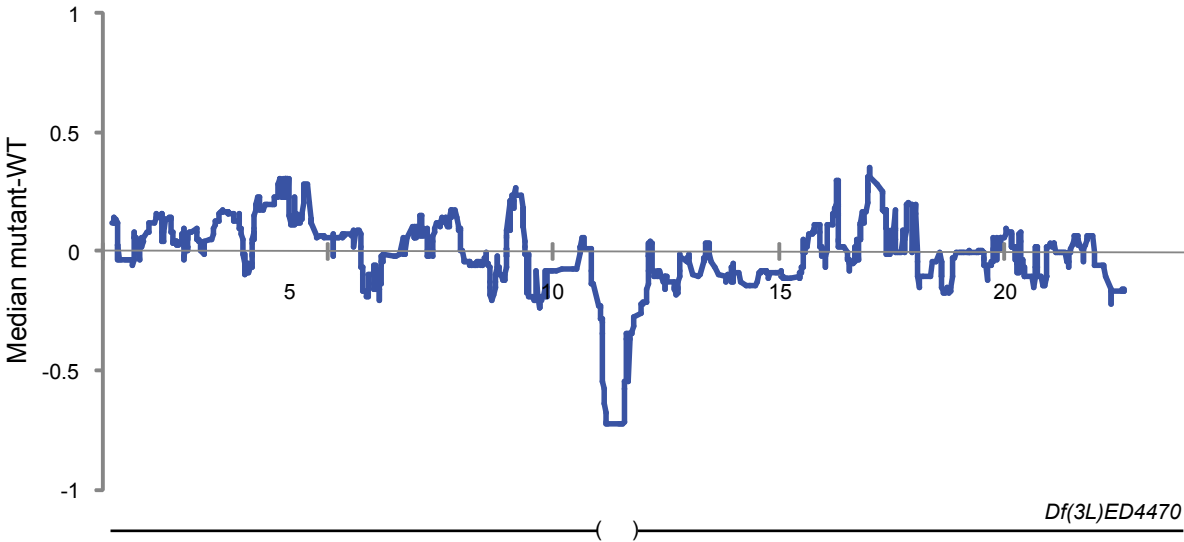

Supplement: Figure S3 — The deficiencies and the duplication mainly affect gene expression within the dose-affected regions. (A) Expression ratios of genes on chromosome 2L in Dp(2;2)Cam3 (green), Df(2L)ED4651 (light blue) and Df(2L)J-H (blue), plotted as moving medians of 41 genes against their positions on the chromosome. (B) Moving medians of gene expression rations in Df(3L)ED4470 against gene position on chromosome 3L. The extents of the aberrations are indicated below each plot. (0.28 MB PDF) [file pgen.1000465.s003.pdf]

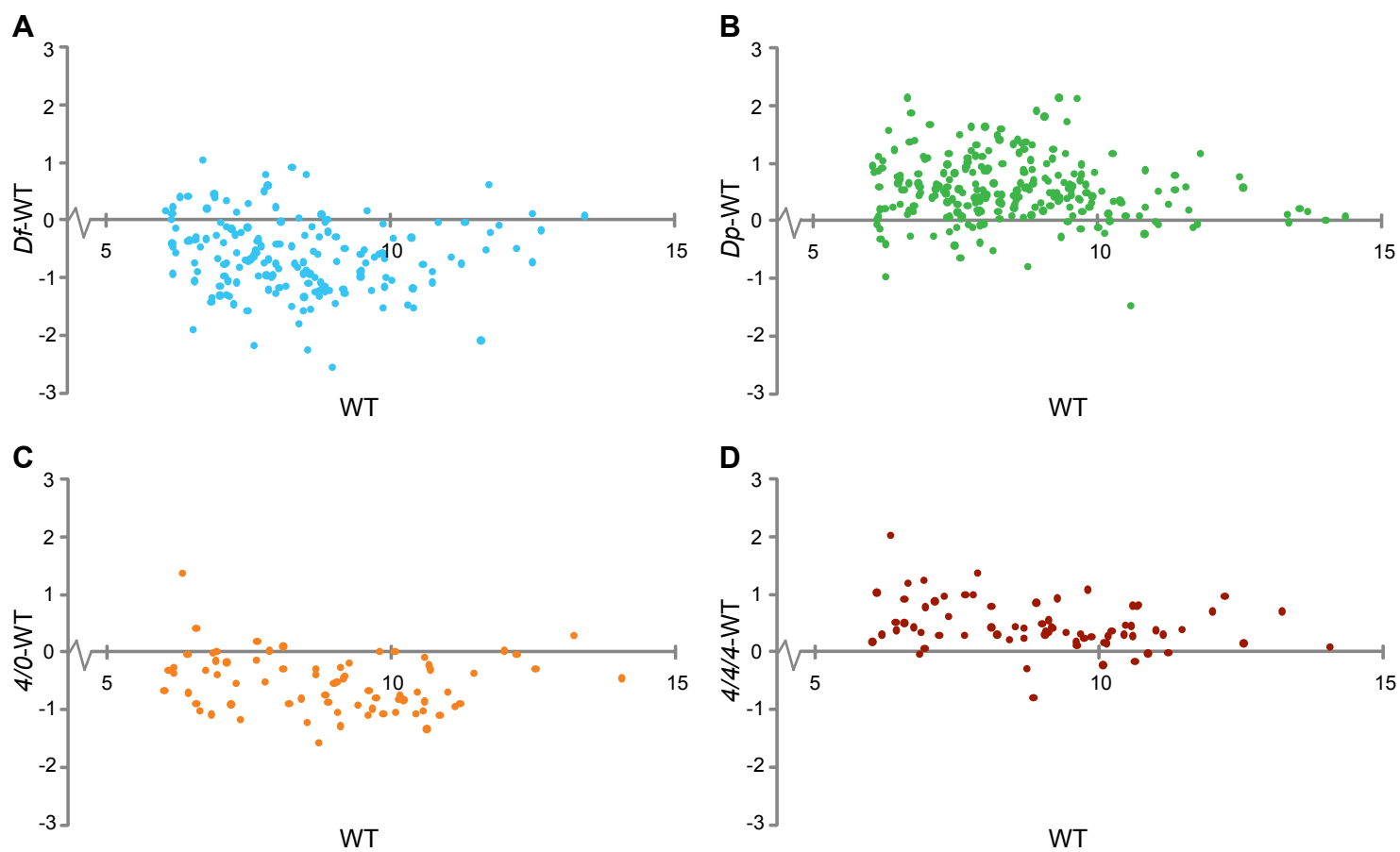

Supplement: Figure S4 — The buffering effect in segmental aneuploidies is not correlated to gene expression levels. Differences in expression levels, plotted as a function of wild type expression levels for all deficiencies - wild type (A), duplication - wild type (B), haplo-4-wild type (C) and triplo-4-wild type (D). (0.12 MB PDF) [file pgen.1000465.s004.pdf]

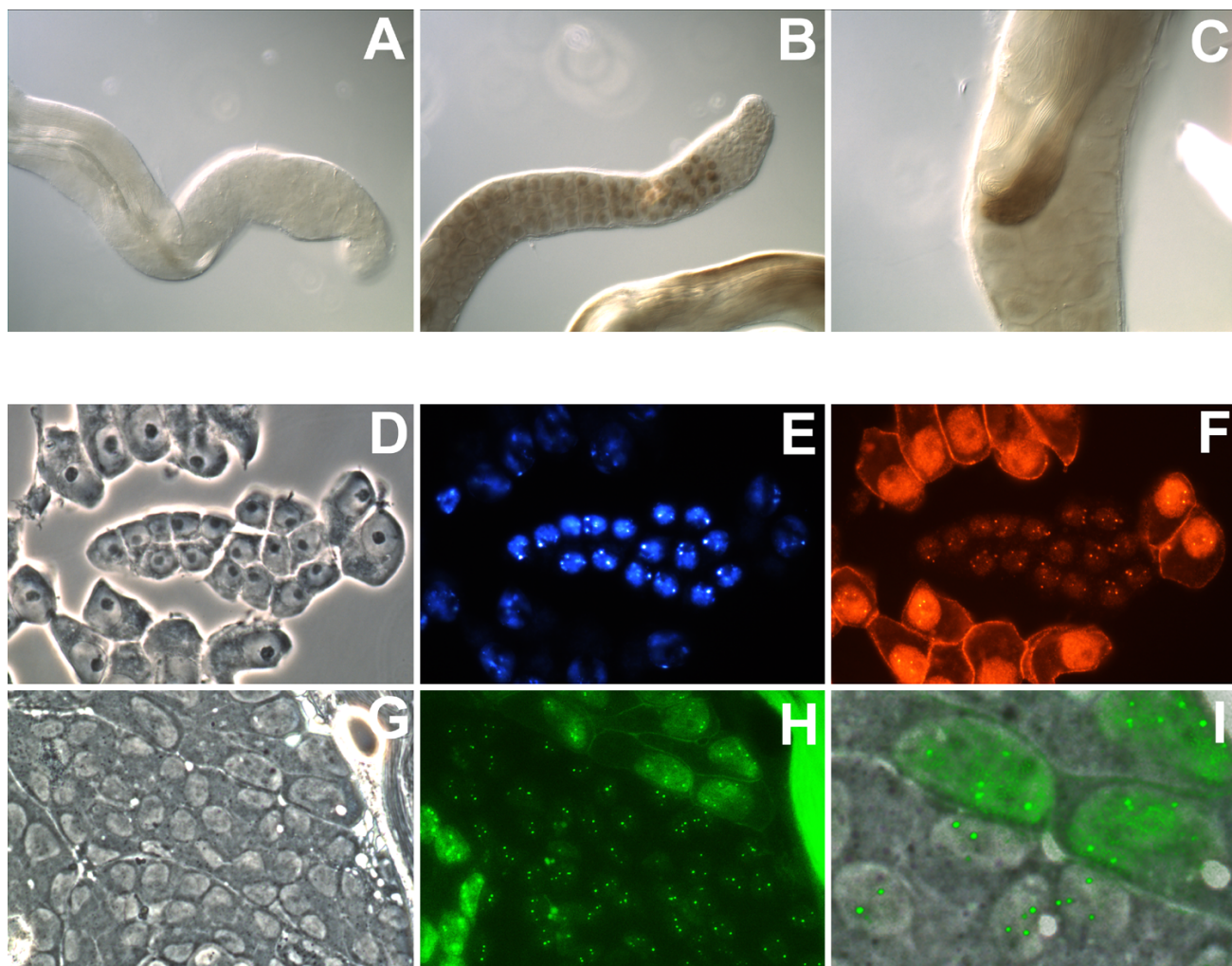

Supplement: Figure S5 — Localisation of POF in testes. (A–C) Whole mount immunostaining of testes preparations. (A) The POF antibody did not detect any signal in the Pof mutant males. (B) POF was detected in young primary spermatocytes, and more strongly in mature primary spermatocytes. (C) POF strongly associates with the nuclear region of the spermatid bundle, and the bundle itself. (D–F) POF strongly associates with 2–4 foci in each nucleus of the 16 young primary spermatocytes. In a later stage of spermatocyte development POF is more evenly distributed. Images taken using phase contrast (D), DAPI (E) and anti-POF antibodies (F). (G–I) Expression of POF.EYFP in unfixed primary spermatocytes, with the young and mature spermatocytes visible in the lower and upper parts of (I), respectively. Note the foci in young spermatocytes and the more dense fluorescence in mature spermatocytes, in accordance with the immunostaining results. Images presented were acquired by phase contrast (G), EYFP fluorescence (H) and merge and zoom (I). The young primary spermatocytes are shown in the lower part of (I) and the more mature spermatocytes in the upper part. (3.64 MB PDF) [file pgen.1000465.s005.pdf]
