# Supplementary material for: Buffering of Segmental and Chromosomal Aneuploidies in Drosophila melanogaster
Source: PLoS Genet. 2009 May 1;5(5):e1000465. doi: 10.1371/journal.pgen.1000465 (PMC2668767; doi:10.1371/journal.pgen.1000465)
Supplement: Table S1 — Genotypes of the flies used in this study and the number of genes before and after the expression cut-off 6. (0.12 MB PDF) [file pgen.1000465.s006.pdf]

Flies (adult females)	Dose	Affected genes	Genes expressed >6	Length
WT	2x			
Df(2L)J-H/+; 4/0	1x	2L:88; 4:105	2L:48; 4:72	~550kb; Entire 4th
Dp(2;2)Cam3/+; 4/4/4	3x	2L:405; 4:105	2L:240; 4:72	~2.5Mb; Entire 4th
Df(3L)ED4470/+	1x	3L:126	3L:58	736kb
Df(2L)ED4651/+	1x	2L:93	2L:60	604kb
Df(2L)J-H/+	1x	2L:88	2L:48	~550kb
4/0	1x	4:105	4:72	Entire 4th
Pof $^{\Delta119}$ (*Pof* $^{-}$)	2x			
Pof $^{\Delta119}$ (*Pof* $^{-}$); *4/4/4*	3x	4:105	4:72	Entire 4th

Testes	Dose	Affected genes	Genes expressed >6	Length
WT	2x			
Pof $^{\Delta119}$ (*Pof* $^{-}$)	2x			
