# Supplementary material for: Buffering of Segmental and Chromosomal Aneuploidies in Drosophila melanogaster
Source: PLoS Genet. 2009 May 1;5(5):e1000465. doi: 10.1371/journal.pgen.1000465 (PMC2668767; doi:10.1371/journal.pgen.1000465)
Supplement: Text S1 — Description and discussion of global effects in expression data and results on POF localisation in testes tissue. (0.02 MB PDF) [file pgen.1000465.s007.pdf]

Supplemental Results and Discussion

Global effects result in skewed distribution

For genome-wide expression studies data handling is important since global effects in the same direction on many genes can skew the results. The standard method recommended by Affymetrix is MAS5. We have not used this method but rather RMA which normalize the data on probe level and not on gene level and will accordingly in theory be less prone to normalize away global effects. It is important to note that if we assume global effects acting on all genes, i.e., a haploid region would cause an increase in expression of all other genes, this effect cannot be measured since we start with a given amount of material (RNA) for labelling. The difference between the haploid region and the rest of the genome can be measured, but whether the remaining genome is globally altered remains unknown. Such global effects could only be measured with one other fixed reference, such as number of cells or amount of DNA and would require that all additional steps, such as labelling, hybridisation and signal read-out are perfectly quantitative. With current methodology global effect acting on all genes cannot be correctly determined.

However, if we assume that a global effect in one direction acts on many (but not all) genes this should cause a skewed distribution. A skewed distribution is a better indicator of global effects than a shift in the distribution. This is exemplified in Figure S1A. To test for global effects, we have calculated the median difference between mutant and wild type using individual probe level raw-data (i.e. prior to any normalisation). We have then looked at the distributions of all our datasets. In all cases small shifts of the entire distribution are detected. These shifts are most probably a consequence of differences in labelling and/or hybridisation. Only in the most extreme case, i.e., the case of *Df(2L)JH/+; 4/0*, we can see a slightly skewed distribution (Figure S1B, black bars). Importantly, this slight skew in the left

tail is mostly accounted for by the probes within the *Df* and within chromosome 4. If these probes are not included (grey bars) we cannot detect a skewed distribution. This doesn't mean that no global effects are present but that the effects are less than the effects of our aneuploidies. Notably, we cannot exclude that small global effects are acting, however, any undetected global effects that might be acting in our experiment will not alter our main conclusion about buffering effect.

POF localisation in testes

It has been shown that the X-chromosome is dosage compensated also in the testes [1]. However, the MSL complex is not involved in dosage compensation of the male germline, and to date the mechanism responsible for compensation in male germline tissues has not been characterized [2,3]. The high expression of POF in the testes and the strong relationship between POF and dosage compensation prompted us to examine whether POF is involved in X-chromosome dosage compensation in the testes. In order to understand the role of POF we performed whole mount staining of wild type male and *Pof* mutant male (*EP(2)2285*) testes. The *EP(2)2285* line has an EP element inserted close to the transcription start point of *Pof* that strongly reduces *Pof* expression, although it does not eliminate it [4]. Accordingly, POF protein was not detected in the testes of *EP(2)2285* mutant males, and while no staining was detected in stem cells close to the testis tip, the primary spermatocyte nuclei were stained in the wild type testes (Figure 5A-5C). Further, dots of staining within the nuclei of young primary spermatocytes were detected, which covered increasingly large areas in increasingly mature samples, until the nuclei of mature primary spermatocytes were completely stained. The sperm bundles were also stained, particularly heavily in the region corresponding to elongated nuclei in the mature sperm bundles (Figure 5C, and further results not shown). To reach a better resolution and separation of different cell types we also analyzed POF

distribution in testes squash preparations. We used both immunofluorescens techniques and *P[Pof.EYFP]* transgenic constructs to confirm the results in live unfixed tissues. The mature primary spermatocyte nuclei were strongly stained in comparison to the diploid cells (Figure 5D-5I), although no POF staining was seen during meiosis or in the first post-meiotic stages prior to the beginning of nuclear elongation (results not shown). In contrast, a few strong foci (2-4) of POF staining were detected in the young primary spermatocytes, and as they grew the number of foci increased and more homogenous staining was detected (Figure 5D-5I). Note that the nucleolus was devoid of POF (Figure 5D and 5F), and that the POF.EYFP fluorescence and antibody-staining signals were nearly identical in the primary spermatocyte and post-meiotic stages (Figure 5 and other results not shown).

The spatial and temporal distribution of POF suggested that in the testes it is first expressed in young primary spermatocytes, in which it is initially attracted to a few nuclear foci of unknown nature. The genome is highly organized in primary spermatocytes and three DAPI-staining domains can be readily identified: two corresponding to the paired metacentric autosomes 2 and 3, and a third corresponding to the sex chromosomes [5,6]. The 4th chromosome is usually believed to be associated with the sex chromosome, but there is no conclusive evidence for this belief and it is possible that the 4th chromosome, which is highly heterochromatic, may be less organised and less localised (like transcribed parts of the Y-chromosome, the “lampbrush”-like loops of which occupy large regions of primary spermatocytes [7,8]). As the primary spermatocytes grow the numbers of foci increased and the distribution of POF became more widespread. However, POF staining was not detected during meiosis or in the first post-meiotic stages, when the spermatids elongate (axoneme elongation) and mature, although it was detected later when the spermatids reached the stage of nuclear elongation. Since transcription is rare or even absent after meiosis these findings indicate that *Pof* RNA and/or protein is/are stored awaiting post-meiotic use.

References

1. Gupta V, Parisi M, Sturgill D, Nuttall R, Doctolero M, et al. (2006) Global analysis of X-chromosome dosage compensation. J Biol 5:3.
2. Rastelli L, Kuroda MI (1998) An analysis of *maleless* and histone H4 acetylation in *Drosophila melanogaster* spermatogenesis. Mech Dev 71:107-117.
3. Bachiller D, Sánchez L (1986) Mutations affecting dosage compensation in *Drosophila melanogaster*: effects in the germline. Dev Biol 118:379-384.
4. Larsson J, Chen JD, Rasheva V, Rasmuson Lestander A, Pirrotta V (2001) Painting of fourth, a chromosome-specific protein in *Drosophila*. Proc Natl Acad Sci U S A 98:6273-6278.
5. Cooper KW (1965) Normal spermatogenesis in *Drosophila*. In: Demerec M, editor. Biology of *Drosophila*. New York: Hafner Publishing Company. pp 1-61.
6. Cenci G, Bonaccorsi S, Pisano C, Verni F, Gatti M (1994) Chromatin and microtubule organization during premeiotic, meiotic and early postmeiotic stages of *Drosophila melanogaster* spermatogenesis. J Cell Sci 107:3521-3534.
7. Bonaccorsi S, Pisano C, Puoti F, Gatti M (1988) Y chromosome loops in *Drosophila melanogaster*. Genetics 120:1015-1034.
8. Hess O (1980) Lampbrush chromosomes. In: Ashburner M, Wright TRF, editors. The genetics and biology of *Drosophila*. London: Academic Press. pp 1-30.
